# Burning the Rat

**DOI:** 10.3201/eid0710.079999

**Published:** 2007-10

**Authors:** Al Zolynas

I find him lying by the door,
legs outstretched as if he died in mid-leap.
I pick him up by the tail.
He feels loose, beyond the first stiffness of death.
His molecules have realized the futility of hanging on;
they know the party's over, it's time to head home.
Suddenly, I want to burn this rat.
I surprise myself at how much I want this.
I want to save him from the slow
decay, the fetid rearrangement
of his parts --or so I tell myself.
But mostly, I want to see him burn.
I drop him on the wire screen
that covers the forty-gallon drum
I use for burning garbage.
I light the fire.
I am strangely satisfied.
As I expected, his whiskers furl
into quick question marks and are gone;
his fur bubbles, then turns black and dry.
The tail, the long nightmare of a tail,
holds on longer than I thought.
Hours later, it is the only thing left,
a white length of ash
like the backbone of something prehistoric
seen from a great distance.

